# Effect and process evaluation of implementing standing desks in primary and secondary schools in Belgium: a cluster-randomised controlled trial

**DOI:** 10.1186/s12966-018-0726-9

**Published:** 2018-09-27

**Authors:** Maïté Verloigne, Nicola D Ridgers, Ilse De Bourdeaudhuij, Greet Cardon

**Affiliations:** 1 Department of Movement and Sports Sciences, Faculty of Medicine and Health Sciences, Ghent University, Watersportlaan 2, 9000 Ghent, Belgium; 2 Research Foundation (FWO), Egmontstraat 1, 1000 Brussel, Belgium; 3 Deakin University, Geelong, Australia, Institute for Physical Activity and Nutrition (IPAN), School of Exercise and Nutrition Sciences, 221 Burwood Hwy, Burwood, VIC 3125 Australia

**Keywords:** Sedentary, Sitting, Bouts, Intervention, Classroom, ActivPAL, Focus group, Questionnaire, Children, Adolescents

## Abstract

**Background:**

Children and adolescents spend a lot of time sitting at school. Implementing standing desks in the classroom is one potential strategy to reduce and break up sitting time. The first aim was to evaluate the effect of implementing standing desks in classrooms in primary and secondary schools on pupils’ sitting-related behaviour and determinants. The second aim was to quantitatively and qualitatively evaluate the process of implementing the desks in the classroom.

**Methods:**

We conducted a cluster-randomised controlled trial with a pre-, mid-, and post-test design including 10 intervention schools (5 primary, 5 secondary schools) and 9 control schools (5 primary, 4 secondary schools) across Flanders, Belgium. Three standing desks were placed in one class in each intervention school for 6 months. At pre-, mid- and post-test, all pupils (*n* = 311; 54.5% girls) completed a questionnaire whilst a subsample of three pupils per class wore an activPAL inclinometer for one school week. Focus groups with pupils and interviews with teachers were conducted at mid-test. Process evaluation questions were added to the mid- and post-test questionnaire for the intervention group. Qualitative data were analysed using NVivo 11. Multilevel regression analyses were conducted in MLwiN 2.31.

**Results:**

Few significant intervention effects were observed, although activPAL data showed favourable intervention effects on primary school pupils’ sitting and standing time and bouts. Focus groups and interviews showed a generally positive attitude towards using standing desks in both teachers and pupils, although some barriers and suggestions for future implementation were noted, for example regarding the amount of desks per classroom. Quantitative process evaluation data showed a low individual use of standing desks (between 57 and 83 min per week), which significantly decreased across the school year for primary school pupils only.

**Conclusions:**

Although pupils and teachers were generally positive about the desks, relatively few intervention effects were found. Future studies should consider how to optimise the use of standing desks in classrooms to impact on sitting time, by for example, determining the most feasible intervention design and by encouraging the continued use of standing desks throughout the school year. Moreover, additional intervention strategies (e.g. educational strategies) might be needed.

**Trial registration:**

NCT03163004. ClinicalTrials.gov. Registered 22 May 2017 (retrospectively registered).

**Electronic supplementary material:**

The online version of this article (10.1186/s12966-018-0726-9) contains supplementary material, which is available to authorized users.

## Background

Sedentary behaviour is defined as “any waking behaviour characterised by an energy expenditure ≤1.5 metabolic equivalents, while in a sitting, reclining or lying posture” [1]. The association between sedentary behaviour and health is less consistent in children than in adults, especially when sedentary behaviour is objectively measured [2–4]. Nevertheless, several studies and reviews have found adverse associations between sedentary behaviour(s) and health in this population, such as unfavourable weight status, reduced physical fitness, and lower self-esteem and school performance [5–8]. Notably, these associations are largely independent from moderate- to vigorous-intensity physical activity [7]. Moreover, since sedentary behaviour in early life tracks into adulthood [9, 10] where it can have potential health implications such as an increased risk of developing type 2 diabetes and metabolic risk factors [6, 11–13], effective strategies are needed to reduce the time that children and adolescents spend in sedentary behaviours. Children and adolescents spend more than 60% of their waking hours sedentary [14–16] of which a large amount is accumulated during school hours whilst sitting in class [17, 18]. Therefore, the classroom is an important setting for implementing specific strategies to reduce sedentary behaviour in this target group [19].

One strategy that has gained attention in recent years is replacing traditional desks and chairs with standing desks [20]. Systematic reviews have summarised the available, albeit limited, evidence regarding the effectiveness of using standing desks. From the findings so far, there is evidence to suggest that standing desks in the classroom can positively affect pupils’ energy expenditure and sitting, standing and stepping time [20–22]. All reviews advocated for more research, as most included studies were pilot studies conducted in one or two schools and had small sample sizes, thus lacking adequate statistical power. In addition, most studies have been conducted in the USA, Australia and New Zealand [23–28], with research from Europe being scarce [25]. Finally, almost all studies have been conducted in primary schools, and only little research in secondary schools [28]. This, arguably, is due to the difference in the school day structure, as pupils from secondary schools generally move to different classrooms throughout the school day whilst primary school pupils tend to remain in one classroom for most lessons. This makes it logistically more challenging to implement standing desks at school (i.e. less exposure to the desks), which may result in more limited intervention effects. In conclusion, more high quality research in a relatively large sample of pupils from primary and secondary schools in a European context is needed to add to the current literature.

With regard to the adoption, success and sustainability of implementing standing desks, it is also important to evaluate how this change in the classroom environment is perceived by both pupils and teachers [20]. Previous studies have found that teachers were positive about the use of standing desks, as this elicited increased space in the classroom and flexibility in learning and improved social interactions and pupils’ motivation concentration and attention [24, 27, 29]. Reported barriers were concerns about the loss of control, increased distraction among pupils, and the standing desks being possibly challenging for less physically active and/or overweight pupils [20, 27]. Focus group research in primary school pupils showed that most children were happy with the standing desks in the classroom, as they made them more alert and focused and facilitated group work and social interaction with teachers [20, 29]. Reported barriers were being tired because of standing, having neck and/or leg pain, and not being able to use the standing desk as other children took their place [20, 29]. A pilot study in which adolescents completed a short process evaluation questionnaire relating to the use of standing desks, found that the majority of respondents enjoyed the lessons more and worked well while standing up; however, about one third reported that they were more distracted and less concentrated [28]. These findings highlight potential benefits and barriers to using standing desks, but more detailed process evaluations are required to further explore teacher and students’ perceptions on the implementation of standing desks in the classroom [21], especially among secondary school teachers and pupils.

Therefore, the first aim of this study was to conduct an effect evaluation of implementing standing desks into the classroom of primary schools (5th Grade) and secondary schools (10th Grade) across Flanders, Belgium using a quasi-experimental design. The 5th Grade was chosen due to children gaining more autonomy and decision-making power regarding health behaviour, thus making this a critical period for changes in health behaviour [30, 31]. In addition, children at this age are able to complete a self-reported questionnaire [32]. The 10th Grade was chosen due to previous research demonstrating a significant increase in objectively-measured sedentary behaviour from early to mid-adolescence [33]. We evaluated the effect on pupils’ sitting time at school, breaks in sitting time at school, determinants of breaking up sitting time, screen-time and school-based factors, assessed by a self-reported questionnaire. In addition, we explored the effect on objectively-measured sitting time, standing time, stepping time, number of sit-stand transitions, and sitting and standing bouts in a subsample of pupils. The second aim was to evaluate the process of implementing standing desks using questionnaire and focus group data from pupils and interview data from teachers. As the school system differs between primary and secondary schools (e.g. home classrooms vs. subject-specific classrooms), differences in the effect and process evaluation between primary and secondary schools were also explored.

## Methods

### Study procedure

This cluster-randomised controlled trial had a pre-, mid- and post-test design and included an intervention and control group. A convenience sample (based on having expressed interest in participating in a standing desks study and being located on a feasible distance of the university) of 26 primary and secondary schools (East- and West-Flanders, Flanders, Belgium) was invited by researchers to participate in the study (June to September 2016). Principals were contacted via email or telephone, and 19 gave permission to participate. The participating schools were randomly assigned to the intervention or control group, stratified by educational system (primary vs. secondary school) and education type (general vs. technical education) by a researcher who was not involved in the recruitment of schools. This resulted in 5 primary and 5 secondary intervention schools and 5 primary and 4 secondary control schools. In each school, the principal was asked to randomly select one class (a 5th Grade class with the majority of pupils being aged 10–11 years old for primary schools and a 10th Grade class with the majority of pupils being aged 15–16 years old for secondary schools). As pupils in Flemish secondary schools often transition between classrooms, principals from secondary schools were asked to select a 10th Grade class that spent at least ten lessons hours per week in one classroom to ensure sufficient exposure to the desks. All children and adolescents in those classes were invited to participate in the study. In total, 343 of the 401 pupils attending the 19 schools provided written (parental) consent (85.5%) and participated in the measurements at the pre-test (November–December 2016), mid-test (February–March 2017) and post-test (May–June 2017). A priori power analysis suggested a sample size of 331 when taking into account a correction factor for the clustering of pupils in schools. After the pre-test, the standing desks were placed in each intervention class for 6 months. The CONSORT checklist for cluster-randomised controlled trials and TIDIER checklist have been completed (Additional files 1 and 2). The study protocol was approved by the ethics committee of the Ghent University Hospital (B670201628738). The trial has been registered at ClinicalTrials.gov (NCT03163004; retrospectively registered on 22 May 2017).

### Implementation of standing desks

The intervention took place from January to June 2017 (end of the school year). Three standing desks (http://www.jaswig.com; 295$ per desk at that time) were added to each intervention class (with a range of 13 to 27 pupils), so no traditional desks were removed from the classroom during the study. The reason to choose three desks is that schools often report the high costs of standing desks as a barrier to implement them [20]. If we aim to broadly promote and scale-up the use of standing desks in schools, practical and less costly solutions are needed (such as a limited amount of desks per class). Moreover, a study that investigated the effect of installing standing desks for all pupils versus using a rotation system with six standing desks found similar effects on children’s sitting time [25]. When the desks were installed, a 10 min PowerPoint presentation was given by a researcher to the teachers to situate our intervention in a health context, based on scientific evidence. We started with defining the health problem (i.e. what is sedentary behaviour), followed by discussing the health consequences of too much sitting and the high prevalence of sitting at school, highlighting that actions or strategies to reduce sitting time at school are needed. This led us to introducing the strategy of implementing standing desks in the classroom. Practical information on the desks was provided as well (e.g. desk height adjustment). All information from the PowerPoint presentation was also provided in a printed teacher manual. Teachers were asked to use a rotation system to make sure that all pupils had equal access to the desks. It was also recommended that pupils rotated from the traditional desks to standing desks approximately every half lesson hour (i.e. every 25 min), however, teachers could adapt this system at their convenience. Further, two posters with the text ‘Standing at your desk every day, keeps the doctor away’ were provided to hang on the classroom wall to motivate/prompt pupils and teachers to use the desks. No other environmental or educational intervention strategies were implemented.

### Effect evaluation measures

#### Questionnaire

All children and adolescents from the intervention and control schools completed a questionnaire in their classroom at pre-, mid- and post-test. As the main goal of the standing desks was to reduce and break up pupils’ sitting time, the questionnaire included two questions on the sitting time and number of breaks in sitting time at school, derived from previously used questionnaires [34, 35]. To explore whether the intervention affected sedentary behaviour at home, we also assessed children’s screen-time during leisure time. We specifically assessed screen-time behaviours (i.e. television watching and compute use) at home, as they are the most prevalent sedentary activities [36]. Also, questionnaires for children and adolescents mostly assess specific sedentary activities and not the general sitting time at home [37] which might be more difficult to estimate and therefore less valid. In addition, determinants of breaking up sitting time were also assessed (i.e. preference, attitude, self-efficacy and habit), derived from the UP4FUN questionnaire within the ENERGY-project [35]. These variables were included as it was hypothesised that an environmental strategy (i.e. introducing the standing desks into the classroom) could affect psychological variables such as self-efficacy, in accordance with the EnRG framework [38, 39]. Finally, school-related variables (i.e. relationship with classmates, involvement in organising school activities and relationship with teachers) were assessed using items from the Flemish Health Behaviour in School-aged Children questionnaire [40]. These variables were included as the additional effects on these school-related variables might be relevant for teachers and school principals in order to convince them to implement standing desks [20]. All questionnaire items and response categories are displayed in Table 1.

**Table 1:** Overview of questionnaire items and response categories

Variable	Questionnaire item	Item(s) handled	Response categories
Effect evaluation measures			
Sitting time at school	*On average, how long do you spend sitting during classes at school per day (do not include study time at home)?*	Single item, recoded into a quantitative variable (min/day) using the midpoint method (e.g., ‘1–2 h’ = 90 min)	1 = None, 2 = 1–15 min/day, 3 = 15–30 min/day, 4 = 30–60 min/day, 5 = 1–2 h/day, 6 = 2–3 h/day, 7 = 3–4 h/day, 8 = 4–5 h/day, 9 = 5–6 h/day, 10 = 6-7 h/day, 11 = More than 7 h/day (recoded into 420 min)
Number of breaks in sitting time at school	*During a normal school lesson, how often do you usually stand up. stretch or walk around a bit?*	Single item, recoded into a quantitative variable (times/h)	1 = Never, 2 = Once, 3 = twice, 4 = three times, 5 = 4 times or more (recoded into 4)
Screen-time	*Roughly how many hours a day do you usually spend watching TV/DVD in your leisure time? (Weekdays and weekend days separately)* *Roughly how many hours a day do you usually use a computer/games console for leisure activities? (Weekdays and weekend days separately)*	Separate items were recoded into quantitative variables (min/day) and following formula was used to assess screen-time: ((TV time + computer time on a weekday)*5) + ((TV time + computer time on a weekend day)*2), divided by 7.	1 = None, 2 = Less than 30 min/day (recoded into 15 min), 3 = 30 min/day, 4 = 1 h/day, 5 = 1 h 30 min/day, 6 = 2 h/day, 7 = 2 h 30 min/day, 8 = 3 h/day, 9 = 3 h 30 min/day, 10 = 4 h or more per day (recoded into 4 h)
Preference to break up sitting time	*I like breaking up the time I spend sitting.*	Single item	1 = Strongly disagree, 2 = Partly disagree, 3 = Neither agree nor disagree, 4 = Partly agree, 5 = Strongly agree
Attitude towards breaking up sitting time	*Breaking up the time I spend sitting is good for me.*	Single item	1 = Strongly disagree, 2 = Partly disagree, 3 = Neither agree nor disagree, 4 = Partly agree, 5 = Strongly agree
Self-efficacy to break up sitting time	*I find it hard to break up the time I spend sitting.*	Single item	1 = Strongly agree, 2 = Partly agree, 3 = Neither agree nor disagree, 4 = Partly disagree, 5 = Strongly disagree
Habit of breaking up sitting time	*Breaking up the time I spend sitting is something that I do without even really thinking about it.*	Single item	1 = Strongly disagree, 2 = Partly disagree, 3 = Neither agree nor disagree, 4 = Partly agree, 5 = Strongly agree
Relationship with classmates	*My classmates like being together* *Most of my classmates are friendly and helpful* *My classmates accept me as I am*	Mean value of three items (Cronbach’s alpha at pre-, mid-, post-test = 0.68, 0.66 and 0.78)	1 = Strongly disagree, 2 = Partly disagree, 3 = Neither agree nor disagree, 4 = Partly agree, 5 = Strongly agree
Involvement in organising school activities	*The pupils in our school are involved in organising school activities*	Single item	1 = Strongly disagree, 2 = Partly disagree, 3 = Neither agree nor disagree, 4 = Partly agree, 5 = Strongly agree
Relationship with teachers	*My classmates and me have good bonds with the teachers at school*	Single item	1 = Strongly disagree, 2 = Partly disagree, 3 = Neither agree nor disagree, 4 = Partly agree, 5 = Strongly agree
Process evaluation measures			
Frequency per week	*How many times per week do you stand at the standing desk (From Monday to Friday)?*	Single item, recoded into a quantitative variable (times/week)	1 = Never (recoded into 0), 2 = Almost never (recoded into 0), 3 = 1 time a week, 4 = 2 times a week, 5 = 3 times a week, 6 = 4 times a week, 7 = once every day (recoded into 5), 8 = more than once every day (recoded into 10)
Duration a time	*If you stand at the standing desk, how long a time do you stand up?*	Single item, recoded into a quantitative variable (min/a time)	1 = 1–15 min a time (recoded into 8 min), 2 = 16–30 min a time or half a lesson hour (recoded into 25 min), 3 = 31–45 min a time (recoded into 38 min), 4 = 46–60 min a time or an entire lesson hour (recoded into 50 min), 5 = more than 1 h a time (recoded into 60 min)
Preference to use the standing desk	*I like taking lessons while standing at the standing desk*	Single item	1 = Strongly disagree, 2 = Partly disagree, 3 = Neither agree nor disagree, 4 = Partly agree, 5 = Strongly agree
Self-efficacy to use the standing desk	*I find it difficult to take lessons while standing at the standing desk*	Single item	1 = Strongly agree, 2 = Partly agree, 3 = Neither agree nor disagree, 4 = Partly disagree, 5 = Strongly disagree
Attitude towards using the standing desk	*Taking lessons while standing at the standing desk is good for me*	Single item	1 = Strongly disagree, 2 = Partly disagree, 3 = Neither agree nor disagree, 4 = Partly agree, 5 = Strongly agree
Habit of using the standing desk	*Taking lessons while standing at the standing desk is something that I do without thinking about it.*	Single item	1 = Strongly disagree, 2 = Partly disagree, 3 = Neither agree nor disagree, 4 = Partly agree, 5 = Strongly agree
Subjective norm related to using the standing desk	*My classmates think it is good that I take lessons while standing at the standing desk*	Single item	1 = Strongly disagree, 2 = Partly disagree, 3 = Neither agree nor disagree, 4 = Partly agree, 5 = Strongly agree

#### activPAL

The teacher of each participating intervention and control class was asked to randomly select three pupils to wear an activPAL monitor (PAL Technologies, Glasgow, UK) for one school week (4 or 5 school days) at the pre-, mid- and post-test. Due to the limited availability of the activPAL monitors to the research team combined with a large number of schools that had measurements in a short period, only a small subsample could be assessed in this study. To have a sufficient amount of valid days, the devices were distributed on Monday or Tuesday and were collected again on Friday afternoon. As the main focus was on school day behaviour, no weekend days were included. The device was attached to anterior mid-line of the right thigh using 3MTM Tegaderm Transparent Film Roll. The activPAL summarises data in 15-s intervals and has shown to be a valid measure for estimating the time spent sitting, standing and walking in this population [41]. The activPAL data were initially downloaded using activPAL3™ software (v7.2.32) and were then processed using a customised Excel macro. Non-wear time was calculated as periods of more than 20 min of consecutive zero counts [42], which is in accordance with accelerometer studies in children [43]. Pupils who provided at least 2 weekdays of a minimum of 9 h wear time per day at each time point were included. The following outcomes were used and/or calculated: (a) sitting time, (b) standing time, (c) stepping time, (d) number of sit-to-stand transitions, (e) frequency of sitting bouts lasting ≥30 min, (f) sitting time accumulated in bouts lasting ≥30 min, (g) frequency of standing bouts lasting at least 1 min, and (h) standing time accumulated in bouts lasting at least 1 min. Sitting and standing bouts were defined as a continuous period of sitting time of at least 30 min or standing/stepping time of at least 1 min, with no tolerance allowed. All outcomes were calculated for an entire weekday and during school hours (8:30 am – 4 pm). The class activities of the included primary and secondary schools started between 8:30 am and 8:40 am and ended between 3:35 pm and 4:20 pm. One start time (8:30 am) and one end time (4 pm) was set for determining the time period during school hours.

### Process evaluation measures

#### Qualitative measures

At the mid-test, focus groups with 6–8 randomly chosen pupils per intervention class (total of 10 focus groups) and an interview with one teacher from each intervention school (total of 10 interviews) were conducted. All focus groups and interviews were recorded and transcribed to facilitate analysis. The interview guide for both pupils and teachers consisted of open-ended questions addressing the following topics: (1) use of standing desks, (2) perceived effects of standing desks, (3) attitude towards using standing desks (including positive and negative aspects), and (4) suggestions for future implementation. It was emphasised that any negative experiences were useful information too. Focus groups lasted about 30 min and interviews about 15 min.

#### Questionnaire for pupils

At the mid- and post-test, process evaluation items were added to the questionnaire for pupils from the intervention group. In total, there were seven questions, including two items on how many times per week (Monday to Friday) they stand at a standing desk and how long they stand at the standing desk each time. The frequency per week and the duration each time were multiplied to calculate the mean duration per week at a standing desk. The five other items were about personal determinants related to taking lessons while standing at a standing desk (i.e. preference, attitude, self-efficacy, habit and subjective norm). All process evaluation questionnaire items and response categories are displayed in Table 1.

### Data analyses

#### Quantitative data

To evaluate the effect of the intervention and to investigate the change in the quantitative process evaluation data, multilevel repeated measures analyses were performed using MLwiN 2.31 (Centre for Multilevel Modelling, University of Bristol, UK). Multilevel modelling (three-level: measurement-pupil-school) took into account the clustering of three measurements for the effect evaluation (pre, mid- and post-test), and two measurements for the process evaluation (mid- and post-test) of pupils in schools (completer analysis). Skewed variables were log-transformed for analyses to improve normality. For ease of interpretation, non-transformed mean values are reported. Pupils’ age and sex were included as a covariates, as they were significantly associated with the main outcome sitting time at school (assessed via the questionnaire). For the activPAL data, wear time was also included as a covariate. For the effect evaluation, the reported ß-value for the interaction effect between ‘time’ and ‘condition’ can be interpreted as the difference in the change in outcome going from pre- to mid-test and from pre- to post-test according to the condition to which pupils belong (intervention vs. control condition). For the process evaluation among pupils, the reported ß-value for the effect of ‘time’ represents the change in outcome going from mid- to post-test in the intervention group. For the effect evaluation using questionnaire data, a three-way interaction effect (condition*time*school) was calculated and reported in the results section. In case of a significant three-way interaction effect for an outcome variable, analyses were stratified by school. Due to the small sample who wore an activPAL and almost all interaction effects with school being significant for the process evaluation, these results are reported separately for primary and secondary school pupils. For all statistical analyses, *p*-values < 0.05 were considered statistically significant; p-values between 0.05 and 0.1 were considered borderline significant. Datasets have been included as Additional files 3 and 4.

#### Qualitative data

To evaluate the process using qualitative data (focus groups and interviews), data were thematically analysed via NVivo 11.0 in several phases [31]. First, a coding scheme was developed based on the focus group and interview guide, consisting of the four main topics. Secondly, a combination was used of both axial coding and inductive coding in order to add other sub-themes that arose in the transcripts to the final coding template [31]. The final coding scheme was then used to code all transcripts. All codes of the transcripts were compared for similarities and variability and interpreted [44].

## Results

### Sample characteristics

Of the 343 pupils who had consent to participate, 322 completed the questionnaire three times, of which 166 were primary school pupils (51.8% intervention group; 54.3% girls; mean age 10.5 ± 0.4 years) and 156 were secondary school pupils (40.3% intervention group; 54.6% girls; mean age 15.5 ± 0.5 years). No differences were found according to age (Beta = 0.04; SE = 1.11), sex (Beta = 0.12; SE = 0.07), and sitting at time at school (Beta = 6.53; SE = 21.54) between the intervention and control condition at baseline. Of those 343 pupils, 57 were asked to wear an activPAL inclinometer at pre-, mid- and post-test. However, at the post-test, pupils from eight schools (*n* = 24) did not wear the inclinometer anymore due to the exam period and/or the very warm weather. Therefore, we only analysed data from the pre- and mid-test. Only 36 pupils had valid data at both time points, of which 18 were primary school pupils (55.6% intervention group, 50.0% girls, mean age 10.5 ± 0.3 years) and 18 were secondary school pupils (55.6% intervention group, 45.0% girls, mean age 15.5 ± 0.3 years).

### Effect evaluation using questionnaire data (intervention and control group)

Results for the total sample can be found in Table 2, including the mean values for the intervention and control group at the pre-, mid- and post-test. Only two (borderline) significant intervention effects were found in the total sample. The relationship with classmates slightly deteriorated for the intervention group and remained stable for the control group between the pre- and post-test. Further, there was a small increase in self-efficacy to break up sitting time for the intervention group and a small decrease for the control group between the pre- and mid-test. The (borderline) significant three-way interaction effects revealed a few differences in intervention effects between primary and secondary school pupils. Table 3 shows the results of the stratified analyses, including the mean values for all groups at the pre-, mid- and post-test. Three significant intervention effects were found for secondary school pupils, but not in the expected direction. The reduction in sitting time at school was larger for the control group than for the intervention group between the pre- and mid-test. The relationship with classmates deteriorated for the intervention group and slightly improved for the control group between the pre- and post-test. Finally, the intervention group reported a decrease for the habit of breaking up sitting time, whereas the control group reported an increase between the pre- and mid-test. For primary school pupils, two intervention effects were found. There was a small increase in self-efficacy to break up sitting time for the intervention group and a decrease for the control group between the pre- and mid-test. Further, the intervention group reported an increase for the habit of breaking up sitting time, whereas the control group reported a decrease between the pre- and post-test.

**Table 2:** Intervention effects on pupils’ questionnaire data

Variabele	PRE Mean (SE)	MID Mean (SE)	POST Mean (SE)	Group x time (Ref: contr x pre) Beta (SE)	Group x time*school (Ref: contr x pre x prim) Beta (SE)
Sitting time at school^a^ (min/day)	I: 334.3 (15.6) C: 332.1 (16.1)	I: 306.0 (15.5) C: 303.8 (16.1)	I: 342.5 (15.5) C: 327.0 (16.1)	Mid: − 0.005(0.030) Post: 0.026(0.030)	**Mid: 0.128(0.059)** Post: − 0.084(0.059)
Number of breaks in sitting time at school (per lesson hour)	I: 2.4 (0.3) C: 2.2 (0.3)	I: 2.2 (0.3) C: 2.2 (0.3)	I: 2.3 (0.3) C: 2.2 (0.3)	Mid: − 0.182(0.144) Post: 0.042(0.145)	Mid: 0.359(0.289) Post: − 0.393(0.289)
Relationship with classmates^a^ (1–5)	I: 4.4 (0.1) C: 4.2 (0.1)	I: 4.4 (0.1) C: 4.2 (0.1)	I: 4.3 (0.1) C: 4.2 (0.1)	Mid: −0.007(0.011) **Post: − 0.024(0.011)**	Mid: − 0.023(0.022) **Post: − 0.041(0.022)**
Involvement in organizing school activities (1–5)	I: 3.8 (0.1) C: 3.6 (0.1)	I: 3.8 (0.1) C: 3.4 (0.1)	I: 3.9 (0.1) C: 3.4 (0.1)	Mid: 0.106(0.147) Post: 0.230(0.147)	Mid: 0.461(0.295) Post: 0.359(0.294)
Relationship with teachers (1–5)	I: 4.1 (0.1) C: 3.6 (0.2)	I: 4.0 (0.1) C: 3.5 (0.2)	I: 4.0 (0.1) C: 3.7 (0.1)	Mid: −0.057(0.122) Post: − 0.137(0.121)	Mid: 0.312(0.244) Post: 0.336(0.243)
Screen-time (min/day)	I: 168.9 (13.9) C: 152.7 (14.8)	I: 165.4 (13.9) C: 155.8 (14.8)	I: 166.5 (13.9) C: 147.6 (14.7)	Mid: −7.722(8.572) Post: 3.144(8.591)	Mid: 19.812(17.116) Post: 12.141(17.147)
Attitude towards breaking up sitting time^a^ (1–5)	I: 4.3 (0.1) C: 4.3 (0.1)	I: 4.3 (0.1) C: 4.2 (0.1)	I: 4.3 (0.1) C: 4.4 (0.1)	Mid: 0.037(0.046) Post: − 0.016(0.046)	Mid: 0.016(0.092) Post: 0.056(0.092)
Preference to break up sitting time (1–5)	I: 3.9 (0.1) C: 3.9 (0.1)	I: 3.9 (0.1) C: 3.9 (0.1)	I: 4.0 (0.1) C: 3.6 (0.1)	Mid: 0.017(0.155) Post: − 0.202(0.155)	Mid: −0.249(0.311) Post: 0.059(0.310)
Habit of breaking up sitting time (1–5)	I: 3.6 (0.1) C: 3.6 (0.1)	I: 3.6 (0.1) C: 3.6 (0.1)	I: 3.6 (0.1) C: 3.5 (0.1)	Mid: −0.043(0.174) Post: − 0.119(0.174)	**Mid: − 0.800(0.349)** ***Post: − 0.682(0.349)***
Self-efficacy to break up sitting time ^a^ (1–5)	I: 3.1 (0.1) C: 3.4 (0.1)	I: 3.2 (0.1) C: 3.1 (0.1)	I: 3.3 (0.1) C: 3.5 (0.1)	***Mid: 0.111(0.064)*** Post: 0.011(0.064)	Mid: −0.163(0.129) ***Post: − 0.233(0.129)***

^a^log-transformed, *I* intervention group, *C(ontr)* control group, *prim* primary school, *SE* standard error

Significant values are indicated in bold

Borderline significant values are indicated in bold and italic

**Table 3:** Intervention effects on pupils’ questionnaire data, stratified for school

		PRE Mean (SE)	MID Mean (SE)	POST Mean (SE)	Group x time (Ref: contr x pre) Beta (SE)
Sitting time at school^a^ (min/day)	Primary school	I: 292.9(17.3) C: 308.4(17.3)	I: 243.2(17.1) C: 294.2(17.2)	I: 315.1(17.1) C: 303.5(17.1)	Mid: − 0.070(0.050) Post: 0.070(0.051)
	Secondary school	I: 375.8 (11.4) C: 362.2(12.5)	I: 366.5(11.4) C: 314.7(12.5)	I: 372.3(11.4) C: 357.6(12.5)	**Mid: 0.058(0.028)** Post: − 0.014(0.028)
Relationship with classmates^a^ (1–5)	Primary school	I: 4.4(0.1) C: 4.3(0.1)	I: 4.4(0.1) C: 4.3(0.1)	I: 4.4(0.1) C: 4.3(0.1)	Mid: 0.003(0.014) Post: − 0.004(0.014)
	Secondary school	I: 4.4(0.2) C: 4.0(0.2)	I: 4.4(0.2) C: 4.1(0.2)	I: 4.2(0.2) C: 4.1(0.2)	Mid: −0.020(0.017) **Post: − 0.045(0.017)**
Habit of breaking up sitting time (1–5)	Primary school	I: 3.5(0.2) C: 3.6(0.2)	I: 3.7(0.2) C: 3.5(0.2)	I: 3.7(0.2) C: 3.4(0.2)	Mid: 0.338(0.244) ***Post: 0.467(0.244)***
	Secondary school	I: 3.7(0.1) C: 3.6(0.2)	I: 3.5(0.1) C: 3.9(0.1)	I: 3.5(0.1) C: 3.5(0.2)	***Mid: −0.456(0.249)*** Post: − 0.216(0.248)
Self-efficacy to break up sitting time ^a^ (1–5)	Primary school	I: 3.2(0.2) C: 3.7(0.2)	I: 3.4(0.2) C: 3.2(0.2)	I: 3.6(0.2) C: 3.6(0.2)	**Mid: 0.188(0.095)** Post: 0.130(0.096)
	Secondary school	I: 3.0(0.1) C: 3.1(0.2)	I: 2.9(0.1) C: 3.1(0.2)	I: 3.0(0.1) C: 3.4(0.2)	Mid: 0.026(0.086) Post: −0.103(0.086)

^a^log-transformed, *I* intervention group, *C(ontr)* control group, *SE* standard error

Significant values are indicated in bold

Borderline significant values are indicated in italic and bold

### Effect evaluation using activPAL data (intervention and control group)

Results (including the mean values for all groups at the pre- and mid-test) are shown in Table 4. The analyses showed some (borderline) significant intervention effects, but mainly in primary school pupils. The primary school intervention group had a decrease in sitting time during school hours, an increase in standing time during school hours and per weekday, a decreased amount of sitting bouts per weekday, a decrease in the time accumulated in those sitting bouts during school hours and per weekday, an increase in the amount of standing bouts during school hours and an increase in the time accumulated in those standing bouts, whereas opposite changes were found for the primary school control group. However, the stepping time per weekday decreased for the primary school intervention group and increased for the primary school control group. Among secondary school pupils, only one borderline significant effect was found for the number of sit-to-stand transitions, with a decrease for the secondary school intervention group and an increase for the secondary school control group.

**Table 4:** Intervention effects on pupils’ activPAL data, separately for primary and secondary schools

		School hours			Entire school day		
		PRE Mean (SE)	MID Mean (SE)	Group x time (Ref: contr x pre) Beta (SE)	PRE Mean (SE)	MID Mean (SE)	Group x time (Ref: contr x pre) Beta (SE)
Sitting time (min)	Primary school	I: 243.8 (8.9) C: 248.1 (8.9)	I: 217.9 (8.9) C: 260.0 (9.1)	**−37.404 (13.323)**	I: 445.8 (13.0) C: 467.2 (13.2)	I: 428.8 (13.1) C: 464.1 (13.5)	−13.569 (15.793)
	Secondary school	I: 302.9 (8.0) C: 307.7 (9.6)	I: 296.9 (9.0) C: 295.2 (10.6)	9.049 (18.349)	I: 571.2 (14.3) C: 594.9 (16.8)	I: 617.4 (15.4) C: 601.0 (19.0)	41.991 (31.285)
Standing time (min)	Primary school	I: 105.6 (7.5) C: 106.1 (7.5)	I: 131.2 (7.5) C: 97.5 (7.6)	**34.148 (9.711)**	I: 195.1 (11.1) C: 188.6 (11.2)	I: 220.5 (11.1) C: 179.8 (11.4)	**34.464 (10.110)**
	Secondary school	I: 73.7 (5.6) C: 66.4 (6.7)	I: 75.4 (6.3) C: 71.7 (7.4)	−4.762 (12.933)	I: 165.9 (10.1) C: 147.4 (11.8)	I: 138.4 (10.8) C: 144.0 (13.4)	−22.854 (22.858)
Stepping time (min)	Primary school	I: 82.5 (7.2) C: 74.8 (7.2)	I: 80.1 (7.2) C: 75.8 (7.3)	−4.426 (7.384)	I: 141.5 (8.3) C: 127.3 (8.4)	I: 134.1 (8.3) C: 138.2 (8.6)	***−18.796 (10.038)***
	Secondary school	I: 49.0 (3.5) C: 51.6 (4.2)	I: 53.4 (3.9) C: 58.8 (4.6)	−4.308 (7.862)	I: 165.9 (10.1) C: 147.4 (11.8)	I: 138.4 (10.8) C: 144.0 (13.4)	−16.087 (11.374)
Number of sit-to-stand transitions	Primary school	I: 43.7 (3.3) C: 42.7 (3.4)	I: 42.7 (3.3) C: 45.7 (3.4)	−4.042 (3.958)	I: 82.7 (5.9) C: 82.8 (6.0)	I: 85.0 (5.9) C: 83.9 (6.1)	1.331 (6.105)
	Secondary school	I: 24.7 (1.8) C: 20.4 (2.1)	I: 21.7 (1.9) C: 21.8 (2.2)	***−5.034 (2.991)***	I: 49.4 (2.7) C: 3.8 (3.2)	I: 47.2 (2.8) C: 46.2 (3.5)	−4.974 (3.980)
Frequency sitting bouts	Primary school	I: 1.5 (0.2) C: 1.6 (0.2)	I: 1.2 (0.2) C: 1.9 (0.2)	−0.578 (0.364)	I: 2.8 (0.3) C: 2.7 (0.3)	I: 2.4 (0.3) C: 3.1 (0.3)	***−0.816 (0.467)***
	Secondary school	I: 3.6 (0.3) C: 3.4 (0.3)	I: 3.6 (0.3) C: 3.1 (0.3)	0.463 (0.545)	I: 6.0 (0.4) C: 5.8 (0.4)	I: 6.5 (0.4) C: 6.3 (0.5)	0.215 (0.773)
Time accumulated in sitting bouts (min)	Primary school	I: 74.4 (9.2) C: 75.9 (9.2)	I: 55.1 (9.2) C: 87.1 (9.6)	***−30.518 (17.245)***	I: 140.4 (14.0) C: 133.6 (14.5)	I: 113.0 (14.2) C: 151.5 (14.9)	***−43.240 (22.743)***
	Secondary school	I: 160.0 (12.8) C: 160.6 (15.3)	I: 164.8 (14.4) C: 147.9 (16.8)	26.073 (26.802)	I: 277.2 (20.1) C: 283.6 (23.6)	I: 312.8 (21.5) C: 314.3 (26.5)	13.014 (41.413)
Frequency standing bouts	Primary school	I: 34.3 (2.0) C: 34.9 (2.0)	I: 37.2 (2.0) C: 31.2 (2.1)	***6.488 (3.378)***	I: 64.5 (3.1) C: 59.8 (3.2)	I: 66.3 (3.2) C: 56.3 (3.3)	5.291 (4.760)
	Secondary school	I: 22.9 (1.7) C: 22.8 (2.0)	I: 21.9 (1.9) C: 22.8 (2.2)	−1.409 (3.681)	I: 46.6 (2.8) C: 45.6 (3.3)	I: 38.7 (3.0) C: 43.3 (3.7)	−5.628 (5.596)
Time accumulated in standing bouts (min)	Primary school	I: 75.3 (7.6) C: 78.8 (7.6)	I: 104.0 (7.6) C: 69.6 (7.7)	**37.648 (9.281)**	I: 142.3 (10.8) C: 139.6 (10.9)	I: 170.9 (10.8) C: 129.0 (11.0)	**39.242 (8.812)**
	Secondary school	I: 60.3 (5.1) C: 53.7 (6.1)	I: 61.8 (5.8) C: 57.4 (6.7)	−2.501 (11.928)	I: 134.8 (9.8) C: 120.5 (11.5)	I: 114.4 (10.6) C: 119.5 (13.0)	−16.751 (21.618)

*I* intervention group, *C(ontr)* control group, *SE* standard error

Significant values are indicated in bold

Borderline significant values are indicated in italic and bold

### Process evaluation using pupils’ questionnaire data (intervention group only)

Process evaluation results are displayed in Table 5. Although the mean values for the process evaluation were observed to be higher for primary school pupils in the intervention group, there were some unfavourable significant time effects in this group. The frequency of using the standing desks, the mean duration at the desk and pupils’ preferences, self-efficacy, and habit to use the standing desk decreased significantly in primary school pupils. Only for the subjective norm to use the standing desks, a borderline significant increase was found. Among secondary school pupils, there was only a borderline significant increase in the frequency of using the desks, and pupils’ attitude towards the desks.

**Table 5:** Change in process evaluation data for primary and secondary school pupils

		MID Mean (SE)	POST Mean (SE)	Time Beta (SE)
Frequency of using the desks ^a^ (times/week)	Primary school	2.94(0.61)	1.80(0.61)	**−0.379(0.093)**
	Secondary school	1.30(0.66)	1.42(0.66)	***0.195(0.104)***
Duration at the desk (min/time)	Primary school	32.35(4.25)	30.18(4.26)	−1.950(1.664)
	Secondary school	41.91(4.94)	41.34(4.95)	−0.580(1.209)
Mean duration at the desk^a^ (min/week)	Primary school	84.31(13.03)	57.69(13.0)	**−0.376(0.113)**
	Secondary school	63.05(30.0)	58.20(29.99)	0.107(0.112)
Preferences to use the desk^a^ (1–5)	Primary school	4.57(0.10)	4.33(0.10)	**−0.093(0.040)**
	Secondary school	3.80(0.17)	3.87(0.17)	0.027(0.040)
Self-efficacy to use the desk^a^ (1–5)	Primary school	4.12(0.15)	3.75(0.15)	**−0.147(0.065)**
	Secondary school	3.09(0.19)	3.33(0.18)	0.085(0.058)
Attitude towards the desk^a^ (1–5)	Primary school	4.48(0.10)	4.39(0.10)	−0.047(0.038)
	Secondary school	3.71(0.13)	3.89(0.13)	***0.057(0.032)***
Habit to use the desk^a^ (1–5)	Primary school	3.59(0.21)	3.03(0.21)	**−0.224(0.078)**
	Secondary school	2.99(0.14)	2.92(0.14)	−0.041(0.074)
Subjective norm to use the desk (1–5)	Primary school	4.11(0.11)	4.32(0.11)	***0.242(0.138)***
	Secondary school	3.73(0.20)	3.70(0.20)	−0.014(0.124)

^a^log-transformed, *SE* standard error

Significant values are indicated in bold

Borderline significant values are indicated in italic and bold

### Process evaluation using qualitative data (intervention group only)

#### Use of the standing desks at school

Some schools used a fixed rotation system (as initially suggested by the researchers), whereas in other schools, pupils had the freedom to use the standing desks whenever they wanted to. When a rotation system was used, it was the teacher who gave the sign to rotate.

*“We always rotate according to a specific scheme, I think that’s easy. And the teacher says when we have to rotate.” (pupil, primary school 5)*

*“A fixed rotation system makes sure it is fair, that every pupil can use the desks.” (pupil, secondary school 2)*

*“It is just: who gets there first, can use the standing desk.” (pupil, secondary school 1)*

*“I first thought, I am going to use a rotation system, but the pupils always come along so well that I actually never had to intervene.” (teacher, primary school 1)*

*“I don’t work with a rotation system anymore, except in the beginning I had to, because they all wanted to stand up so then they had to take turns.” (teacher, secondary school 1)*

In most schools (especially in secondary schools), pupils stood at the desk for an entire lesson hour, as they do not have to keep an eye on the time then. In some primary schools, pupils stood up for half a lesson hour (which was initially suggested by the researchers).

*“You’re always busy during the lessons and you don’t look at the clock, so that’s why we rotate every hour and not every half hour.” (pupil, secondary school 4)*

*“We are always with three pupils who can stand up for half a lesson hour, and then we rotate.” (pupil, primary school 2)*

*“Yes, I try to keep an eye on my watch. Pupils stand at the desk for let’s say half an hour and then we switch.” (teacher, primary school 5)*

Some pupils and teachers indicated that the desks were more used in the beginning. In one secondary school, the desks were not used anymore at the end of the school year.

*“It used to be special, but now we often forget.” (pupil, primary school 1)*

*“In the beginning there was a lot of enthusiasm, but then it might have decreased.” (teacher, secondary school 1)*

*“In the beginning it actually went well, the desks were used. But then it started to slack off and we are now at the point that they are actually not used anymore.” (teacher, secondary school 5)*

*“It is mainly the same pupils who keep using the standing desks.” (teacher, secondary school 3)*

### Perceived effects of using the standing desks

The large majority of pupils in primary schools thought that they could concentrate better when they were standing at the desk. This was also reiterated by their teachers. Some pupils explained that this was because they talked less with classmates or fidgeted less.

*“I go to a speech therapist for my concentration. It is better at a standing desk. I can concentrate more.” (pupil, primary school 4)*

*“In general, the concentration at those desks is very good. Of course, it depends on who they are standing next to.” (teacher, primary school 3)*

In secondary schools, opinions were more divided on the effect on concentration.

*“I don’t think you can concentrate better, but it’s not that I lose my concentration.” (pupil, secondary school 4)*

*“I generally notice that they can concentrate better. Pupils who mostly not give a lot of answers, will now answer more quickly, that is something that I notice.” (teacher, secondary school 3)*

*“There is a loss of concentration, because they feel like ‘I am standing here and I don’t have to do anything’.” (teacher, secondary school 5)*

Pupils from both primary and secondary schools perceived that they had a better posture when standing at the desk. Other perceived effects from pupils included feeling less tired and more energetic, although this was noted more frequently by pupils from primary schools. Pupils from secondary schools generally perceived fewer other effects.

*“If you sit at a regular desk, you are writing with your head down, but at the standing desks you write with a straight back.” (pupil, primary school 4)*

*“If you sit down, you will quickly sag and sit down with a pleated back. If you stand up, you pay more attention to stand up correctly.” (pupil, secondary school 4)*

*“You sometimes feel less tired after a lesson where you have stood up.” (pupil, secondary school 2)*

Some secondary school pupils said that they did not perceive any effects because they did not spend sufficient time at a standing desk.

*“I think that it can make a difference on the long term, and not immediately after a lesson.” (pupil, secondary school 2)*

*“I think that we stand up too little to feel an effect.” (pupil, secondary school 3)*

### Attitude towards using standing desks

Most pupils and teachers were generally positive about using standing desks in the classroom. Standing desks were thought to be fun, a good idea and a good initiative. The most important advantage of the standing desks was being able to switch between sitting and standing throughout the day. Other advantages were the usability of the desks and the improved posture of pupils.

*“I think a standing desk is better, because otherwise you almost sit all the time, about 7 hours a day, now you have a bit more physical activity, and I like physical activity.” (pupil, primary school 4)*

*“I think it’s good like this. Standing up a bit and then sitting down a bit.” (pupil, primary school 5)*

*“It is nice that if you want to stand up some time, you can actually do that, instead of really having to keep sitting down.” (pupil, secondary school 2)*

*“I am really a fan. I am all for more physical activity anyhow, so I also try to incorporate that in my lessons.” (teacher, primary school 1)*

*“The fact that they stand up might not be so bad, also for their health. By standing up they are less attached to their chair and they move more than when they sit on a chair.” (teacher, secondary school 1)*

Some teachers from secondary schools noticed differences between pupils in how much they like to stand up during lessons.

*“Some pupils like to stand, that is something I see, others like it less. That’s typical for adolescents.” (teacher, secondary school 3)*

*“Some pupils felt like ‘Do we still have to stand up, we don’t think it’s pleasant after all’.” (teacher, secondary school 5)*

The fact that the desks were placed at the back of the class came with both advantages and disadvantages. Some pupils were able to see the front of the class, whilst others found it difficult to see. In addition, the larger distance between the teachers and the pupils at the standing desks encouraged some pupils to copy during tests. In addition, it was sometimes mentioned by both pupils and teachers that standing up at the desks might not be feasible for all lessons or lesson contents.

*“I think it’s good because in the back you have a good overview and you can see the blackboard very well.” (pupil, primary school 1)*

*“If it is needed for you to pay attention, and you are at the back and you don’t get involved, then, yeah, you don’t want to stand there.” (pupil, secondary school 4)*

*“If it is a really important lesson, I still let them sit down, because the pupils can then sit closer to the blackboard. Otherwise they’re too far away.” (teacher, primary school 4)*

*“The only thing I do not allow is to do a test at the desks, because it was too easy to copy.” (teacher, primary school 1)*

Both groups also noted some other barriers or difficulties, although this was generally more the case in the secondary schools. In general, some teachers indicated that it was less practical to implement three extra standing desks in the set-up of smaller classrooms.

*“We don’t really have the accommodation for that, so yeah, the only option was right at the back of the classroom.” (teacher, primary school 1)*

*“I don’t think it is optimal with 27 in that small classroom. Because they are already sitting on each other and then those desks have to be there too.” (teacher, secondary school 1)*

Furthermore, some pupils and teachers from secondary schools indicated it would have been more easy if they had already used standing desks from primary school.

*“It is taking some time getting used to. Because you did not know how to stand there.” (pupil, secondary school 4)*

*“It is not a habit that they have. Maybe if it would already have been learned from primary school, it might feel more natural for them. So that they know, look, every once in a while we stand up.” (teacher, secondary school 3)*

Some teachers from secondary schools mentioned that it could create chaos in the classroom. One teacher from a secondary school said that it could be more difficult for older or more traditional teachers to have standing desks in their classroom.

*“The disciplinary aspect. We always say to our students ‘First stand up behind the chair and if it is quiet, take a seat.’ That is something that doesn’t work if you would have an entire classroom with standing desks. Sitting down actually can bring some rest.” (teacher, secondary school 1)*

*“I have heard colleagues who said ‘Those desks are not handy!’, but that were mostly older and more traditional teachers who are not open to that.” (teacher, secondary school 1)*

*“But of course, I am open for that. I think that as a teacher, if you are negative about it, it would be different and you would pass this on to the pupils (…) You have to be open-minded, not everyone is like that.” (teacher, primary school 5)*

In one secondary school, pupils did not feel really supported from the teachers to use the standing desks.

*“If the teachers would cooperate more, we would use it more often.” (pupil, secondary school 5)*

### Suggestions for future implementation

A lot of pupils suggested installing standing desks in other classrooms/schools. In addition, all teachers but one would recommend them to other teachers or to other schools. Suggestions for others were to take into account pupils’ preferences and to pay attention to the class organisation.

*“I would definitely recommend them. It creates a vivid moment in the classroom.” (teacher, primary school 5)*

*“I am now not really convinced of the added value.” (teacher, secondary school 5)*

Both pupils and teachers from primary schools would only implement it from the older years.

*“I would do it for the 4* ^*th*^ *, 5* ^*th*^ *and 6* ^*th*^ *grade, I think pupils from the first and second grade would get more tired if they stand up at a standing desk (…) I think it is a bit too difficult for them.” (pupil, primary school 4)*

Almost all pupils and teachers from primary and secondary schools indicated that three standing desks were not sufficient, as only three pupils can stand up at the same time. Some suggested to implement four or five standing desks per classroom, others suggested to replace half of the sitting desks with standing desks.

*“I would install some more desks, so more children can stand up and also for a longer time.” (pupil, primary school 4)*

*“Maybe fifty-fifty, half of the class sits down, half of the class stands up, then they can switch more often.” (teacher, primary school 1)*

*“Let’s say in an average class of 20 pupils, I would place five desks. One fourth of the class.” (teacher, secondary school 3)*

Finally, according to the teachers the most important barrier for schools to implement standing desks would be the high price.

*“The price, that’s a no-go… I think that is very unfortunate.” (teacher, primary school 2)*

*“If you spend a lot of money on such a desk, knowing that pupils will draw on it or stick gum to it…. I think that is the biggest disadvantage, because the fact that they stand up, that’s nothing but an advantage I think.” (teacher, secondary school 3)*

## Discussion

This study evaluated the effect and process of implementing standing desks in the classroom of primary and secondary schools across Flanders, Belgium. In general, very few significant intervention effects were found, however, the activPAL data revealed some positive intervention effects for primary school pupils only. In addition, the process evaluation showed that most pupils and teachers were generally positive about using standing desks in the classroom, though some potential barriers and concerns were noted.

There are several possible reasons for the general lack of effect observed. One reason could be the limited amount of standing desks in each classroom, restricting the amount of time that pupils could spend at the standing desk (on average 60 min per week) and potentially reducing the chance to create a real habit for standing. In addition, our process evaluation showed that the use largely varied between schools and pupils. Previous studies have often replaced all traditional desks with standing desks (including a stool) [20], yet most of them did not provide specific information on how many minutes per day or per week pupils actually stood at the standing desks [20–22]. A previous study that compared the effect of installing standing desks for all pupils versus using a rotation system with less standing desks, found similar effects on children’s sitting time [25]. However, there were still six standing desks available and children were exposed to them at least 1 h per day, which is far more than our average of 60 min per week. To the best of our knowledge, the current study installed the smallest number of standing desks in the classroom compared to other studies. In the focus groups and interviews, pupils and teachers suggested to install more than three desks, with suggestions ranging from four or five to half of the class. Nevertheless, it remains difficult to recommend how many desks are needed, especially when considering the high costs and the limited classroom space, which was mentioned several times by teachers in the interviews. Alternative desk models might be explored, such as high tables that can accommodate several pupils. Further, it might even not be relevant to install a larger amount, as both the qualitative and quantitative process evaluation showed a decreased use of the desks throughout the school year, although this might also be due to the inability of creating ‘a standing habit’ with only three desks. Some pupils and teachers indeed indicated in the focus groups and interviews at the mid-test that the desks had higher use in the beginning. The quantitative data confirmed this for primary school pupils, as there was a significant decrease in the frequency of using the desks from the mid- to post-test, resulting in a decreased duration per week at the desk. In addition, preferences, self-efficacy and even habit of using standing desks decreased in this group, although it could be observed that the mean values for primary school pupils were still generally the same or even higher at the post-test than those for secondary school pupils. In the literature, it has been stated that introducing standing desks may stimulate a novelty effect [22, 45]. Thus, in the future it should be determined what the most effective and feasible intervention design is and how the continued use of standing desks throughout the school year(s) can be encouraged. At the same time, it is also important to offer teachers and pupils the choice on how to use the desks, by for example, providing pedagogical training.

A second potential reason for the general lack of effect is the use of a questionnaire to examine intervention outcomes. The reason to use a questionnaire was twofold: it allowed us to measure a relatively large sample at three time points and allowed us to investigate the effect on outcomes such as screen-time, determinants of breaking up sitting time, and school-related variables, which are generally assessed by a questionnaire. Investigating these outcomes might be important for starting to examine the broader impact of standing desks. However, questionnaires are subjected to recall bias and social desirability and generally have lower reliability and validity compared to objective measures [46]. Further, the items in our questionnaire were derived from previous questionnaires [34, 35, 40], and might have been less appropriate for a standing desks intervention. It could therefore be relevant to develop a questionnaire that is more specifically related or tailored to the implementation of standing desks in schools and that is sufficiently sensitive to capture changes due to the intervention. Of course, using an objective posture monitor provides accurate and relevant data and is still recommended. In this study, we found more (positive) intervention effects when using activPAL data. However, only a subsample of pupils was asked to wear an activPAL for practical reasons, and few pupils wore the monitor at the last time point, leaving us with only a very small sample that could be analysed between the pre- and mid-test. These results should therefore be interpreted with caution. Previous studies evaluating the effect of standing desks in schools via activPAL, also measured relatively small samples [25–29]. Thus, future studies should use activPAL monitors in a large sample of pupils - although being aware of issues reported in relation to participant burden [47, 48] -, in combination with a questionnaire that is specifically related to sitting and standing time at school and to the implementation of standing desks in schools. One other possibility might be to use self-reported diaries or logs and external observers to assess pupils’ sitting and standing time [49].

A final reason for the general lack of effects could be that no other environmental or educational strategies were implemented in this study [21, 22]. A study conducted in Australia combined both standing desks as well as pedagogical strategies to reduce and break up children’s sitting time in primary school and found favourable effects on their sedentary patterns [26]. Silva and colleagues [50] investigated the effected of an intervention combining standing desks with teacher training and educational and motivational sessions with students and parents and also found significant improvements for sitting and standing time. Future studies should investigate if it is more effective to implement extra intervention strategies, such as educational components, goal setting techniques and pedagogical training [20], rather than providing an environmental change to the classroom alone.

Although there was a general lack of intervention effect observed overall, some differences were evident between primary and secondary schools. For primary schoolchildren we only found two small intervention effects when looking at the questionnaire data (i.e. self-efficacy to break up sitting time and habit of breaking up sitting time), but several intervention effects when looking at the activPAL data (notwithstanding the small subsample). For example, the sitting time during school hours (− 26 min) and total time spent in sitting bouts during school hours (− 19 min) and across the whole school day (− 27 min) decreased in the intervention group and increased in the control group (+ 12 min, + 11 min, + 18 min, respectively), whereas the standing time and total time spent in standing bouts during school hours (+ 26 min, + 29 min, respectively) and across the whole school day (+ 25 min, + 29 min, respectively) increased for the intervention group and decreased for the control group by about 10 min. It is encouraging that the majority of intervention effects remained significant when focusing on the whole day. However, primary school pupils’ stepping time slightly decreased for the intervention group (− 7 min) which could suggest they compensate in their physical activity [45]. Although our results with regard to questionnaire data cannot be compared to other studies as these outcomes have not been investigated before, the activPAL results are in accordance with other standing desks studies in primary school children that used an activPAL [25–27, 29, 50]. These studies also found (significant) increases in standing time and (significant) decreases in sitting time during class time and/or over the school day, although the relative change was mostly larger in those studies. Some of them found significant increases in stepping time as well [25, 29], which is different from our results. For secondary school pupils, of the few intervention effects that were found using questionnaire data, they were not in the expected direction and there were generally no intervention effects with regard to the activPAL data. A possible explanation for the difference is that primary school pupils often have one main teacher and their own classroom, which might improve both the practicality and the accessibility of the intervention [22]. Adolescents often transition between school classrooms for different lessons, however, for most school it is financially not possible to install standing desks in all those classrooms, resulting in limited exposure to standing desks. Indeed, our quantitative process evaluation data showed a lower mean duration at the standing desk per week in secondary school pupils compared to primary school pupils (especially at the mid-test). Additionally, the difference in the nature of lessons between primary and secondary schools might also have an impact. For example, the lessons in primary schools which are often more playful and interactive could lend themselves more for using the standing desks. Previous standing desks interventions were mostly conducted in primary schools, probably for that reason [20, 22], however, a previous study in which adolescents also had a low exposure to the standing desks (1–2 lessons per week) found a positive effect on adolescents’ sitting and standing time and bouts [28]. Nevertheless, this pilot study included a small study sample, so more research in adolescents is needed. In addition, the authors hypothesised that because of the low exposure to the desks, some pupils experienced some problems with adjusting and getting used to the desks, which is important to take into account when implementing standing desks in secondary schools [28]. Similarly, it might be hard to adjust for secondary school teachers who often move between different classrooms as well, suggesting they might be more difficult to get on board compared to primary school teachers. These assumptions should be further investigated and future studies need to consider how standing desks are ideally incorporated in the secondary school system.

Despite the lack of intervention effects, pupils and teachers were generally positive about the standing desks, which is in line with the findings of previous studies [20, 24, 51]. Teachers underlined the importance of letting children and adolescents break up their sitting time and stand up once in a while, and pupils enjoyed being able to transition between sitting and standing. Both pupils and teachers perceived an effect on pupils’ concentration level, although this was primarily the case among primary school pupils. In secondary schools, these perceptions were mixed. Therefore, it would be relevant to objectively assess the effect of standing desks on specific cognitive or academic performances, as the evidence is currently scarce [20–22]. Only two studies have already assessed the effect of standing desks on pupils’ cognitive function with preliminary evidence for positive effects, but the small sample size and/or lack of control group [49, 52] suggest more research is needed. This information would arguably be more important for schools than any potential health benefits [20]. More specifically, if strong evidence for beneficial or at least no detrimental effects on pupils’ cognitive, learning or academic performances are found, this might persuade all teachers (including the more conservative ones) to use them in the classroom. Indeed, our qualitative findings showed that not all teachers are convinced of the added value, which is important for an optimal implementation. Finally, specific attention should be given on how to organise the standing desks in the classroom set-up in order to have sufficient space and to avoid pupils at the desks being able to copy during tests or feel isolated from the rest of the class.

Study strengths were (a) the evaluation of both the effect and process (based on qualitative and quantitative data) of implementing standing desks, (b) the relatively large study sample across 19 classes from both primary and secondary schools in a European context, and (c) the study design with three measurements using an intervention and control condition. These strengths build upon the research conducted so far. The main limitation relates to the effect measures, that is the use of a questionnaire and the small sample providing (valid) activPAL data. A second limitation is that the focus groups and interviews were only conducted at the mid-test, due to the fact that the end of the school year is a busy period for schools (e.g. exams). However, as the quantitative process evaluation data showed, for example, a decrease in the frequency of using the standing desks in primary schools, it would be relevant to conduct a qualitative process evaluation at the end of the school year as well. Another limitation is related to the impact of teachers on the effects of the standing desks. Although we took into account the class-level in our multilevel analyses, we were not able to control our analyses for potential influence of the teachers. Teachers are important “agents” of pupils’ sedentary behaviour, suggesting future interventions could specifically focus on changing important determinants, such as teachers’ attitude and self-efficacy towards using standing desks. Finally, although a cluster-randomised controlled trial was conducted, some methodological issues need to be acknowledged, such as the selection bias which could have occurred when principals selected one specific class in their school to participate in the study and when teachers selected three pupils to wear an activPAL. In addition, the convenience sampling of schools might have led to a lack of representativeness to the wider population. Indeed, schools were only located in two provinces in Flanders (with the majority in East-Flanders) and almost 80% of secondary schools exclusively offered general education, suggesting the findings of our study cannot be generalised to the entire (secondary) school population.

## Conclusion

This study on implementing standing desks into the classroom of Flemish primary and secondary schools found that most pupils and teachers were generally positive about using standing desks in the classroom. However, there were generally not many or large intervention effects, especially when questionnaire data were analysed. Some favourable intervention effects were found on primary school pupils’ objectively measured sitting and standing time and bouts, but this was based on data from a small subsample. Future studies should determine the most effective and feasible intervention design and how the continued use of standing desks throughout the school year(s) can be encouraged. Further, it should be investigated if additional intervention strategies are required for behavioural change. To assess the effect, future studies should use activPAL inclinometers in a large sample of pupils, possibly in combination with a questionnaire that is specifically related to sitting and standing time at school and to the implementation of standing desks in schools.

## Additional files

Additional file 1: CONSORT checklist for cluster-randomised controlled trials. (PDF 375 kb)

Additional file 2: TIDieR checklist. (PDF 429 kb)

Additional file 3: Dataset questionnaire. (SAV 134 kb)

Additional file 4: Dataset activPAL. (SAV 21 kb)

## Abbreviations

C: Control group
I: Intervention group
SE: Standard error

## References

[1] Tremblay MS, Aubert S, Barnes JD, Saunders TJ, Carson V, Latimer-Cheung AE, Chastin SFM, Altenburg TM, Chinapaw MJM (2017). Sedentary behavior research network (SBRN) - terminology consensus project process and outcome. The international journal of behavioral nutrition and physical activity.

[2] van Ekris E, Altenburg TM, Singh AS, Proper KI, Heymans MW, Chinapaw MJ (2016). An evidence-update on the prospective relationship between childhood sedentary behaviour and biomedical health indicators: a systematic review and meta-analysis. Obesity reviews : an official journal of the International Association for the Study of Obesity.

[3] Chinapaw M, Altenburg T, Brug J (2015). Sedentary behaviour and health in children - evaluating the evidence. Prev Med.

[4] Cliff DP, Hesketh KD, Vella SA, Hinkley T, Tsiros MD, Ridgers ND, Carver A, Veitch J, Parrish AM, Hardy LL (2016). Objectively measured sedentary behaviour and health and development in children and adolescents: systematic review and meta-analysis. Obesity reviews : an official journal of the International Association for the Study of Obesity.

[5] Carson V, Hunter S, Kuzik N, Gray CE, Poitras VJ, Chaput JP, Saunders TJ, Katzmarzyk PT, Okely AD, Connor Gorber S (2016). Systematic review of sedentary behaviour and health indicators in school-aged children and youth: an update. Applied physiology, nutrition, and metabolism = Physiologie appliquee, nutrition et metabolisme.

[6] de Rezende LF, Rodrigues Lopes M, Rey-Lopez JP, Matsudo VK, Luiz Odo C (2014). Sedentary behavior and health outcomes: an overview of systematic reviews. PLoS One.

[7] Mitchell JA, Pate RR, Beets MW, Nader PR (2013). Time spent in sedentary behavior and changes in childhood BMI: a longitudinal study from ages 9 to 15 years. International journal of obesity (2005).

[8] Salmon J, Tremblay MS, Marshall SJ, Hume C (2011). Health risks, correlates, and interventions to reduce sedentary behavior in young people. Am J Prev Med.

[9] Biddle SJ, Pearson N, Ross GM, Braithwaite R (2010). Tracking of sedentary behaviours of young people: a systematic review. Prev Med.

[10] Hancox RJ, Milne BJ, Poulton R (2004). Association between child and adolescent television viewing and adult health: a longitudinal birth cohort study. Lancet (London, England).

[11] Proper KI, Singh AS, van Mechelen W, Chinapaw MJ (2011). Sedentary behaviors and health outcomes among adults: a systematic review of prospective studies. Am J Prev Med.

[12] Thorp AA, Owen N, Neuhaus M, Dunstan DW (2011). Sedentary behaviors and subsequent health outcomes in adults a systematic review of longitudinal studies, 1996-2011. Am J Prev Med.

[13] van der Ploeg HP, Chey T, Korda RJ, Banks E, Bauman A (2012). Sitting time and all-cause mortality risk in 222 497 Australian adults. Arch Intern Med.

[14] Colley RC, Garriguet D, Janssen I, Craig CL, Clarke J, Tremblay MS (2011). Physical activity of Canadian adults: accelerometer results from the 2007 to 2009 Canadian health measures survey. Health Rep.

[15] Steele RM, van Sluijs EM, Cassidy A, Griffin SJ, Ekelund U (2009). Targeting sedentary time or moderate- and vigorous-intensity activity: independent relations with adiposity in a population-based sample of 10-y-old British children. Am J Clin Nutr.

[16] Verloigne M, Van Lippevelde W, Maes L, Yildirim M, Chinapaw M, Manios Y, Androutsos O, Kovacs E, Bringolf-Isler B, Brug J (2012). Levels of physical activity and sedentary time among 10- to 12-year-old boys and girls across 5 European countries using accelerometers: an observational study within the ENERGY-project. The international journal of behavioral nutrition and physical activity.

[17] Abbott RA, Straker LM, Mathiassen SE (2013). Patterning of children's sedentary time at and away from school. Obesity (Silver Spring, Md).

[18] van Stralen MM, Yildirim M, Wulp A, te Velde SJ, Verloigne M, Doessegger A, Androutsos O, Kovacs E, Brug J, Chinapaw MJ (2014). Measured sedentary time and physical activity during the school day of European 10- to 12-year-old children: the ENERGY project. J Sci Med Sport.

[19] Salmon J (2010). Novel strategies to promote children's physical activities and reduce sedentary behavior. J Phys Act Health.

[20] Hinckson E, Salmon J, Benden M, Clemes SA, Sudholz B, Barber SE, Aminian S, Ridgers ND (2016). Standing Classrooms: Research and Lessons Learned from Around the World. Sports medicine (Auckland, NZ).

[21] Minges KE, Chao AM, Irwin ML, Owen N, Park C, Whittemore R, Salmon J (2016). Classroom standing desks and sedentary behavior: a systematic review. Pediatrics.

[22] Sherry AP, Pearson N, Clemes SA (2016). The effects of standing desks within the school classroom: a systematic review. Preventive medicine reports.

[23] Benden ME, Blake JJ, Wendel ML, Huber JC (2011). The impact of stand-biased desks in classrooms on calorie expenditure in children. Am J Public Health.

[24] Blake JJ, Benden ME, Wendel ML (2012). Using stand/sit workstations in classrooms: lessons learned from a pilot study in Texas. Journal of public health management and practice : JPHMP.

[25] Clemes SA, Barber SE, Bingham DD, Ridgers ND, Fletcher E, Pearson N, Salmon J, Dunstan DW (2016). Reducing children's classroom sitting time using sit-to-stand desks: findings from pilot studies in UK and Australian primary schools. J Public Health (Oxf).

[26] Contardo Ayala Ana, Salmon Jo, Timperio Anna, Sudholz Bronwyn, Ridgers Nicola, Sethi Parneet, Dunstan David (2016). Impact of an 8-Month Trial Using Height-Adjustable Desks on Children’s Classroom Sitting Patterns and Markers of Cardio-Metabolic and Musculoskeletal Health. International Journal of Environmental Research and Public Health.

[27] Hinckson EA, Aminian S, Ikeda E, Stewart T, Oliver M, Duncan S, Schofield G (2013). Acceptability of standing workstations in elementary schools: a pilot study. Prev Med.

[28] Sudholz B, Timperio A, Ridgers ND, Dunstan DW, Baldock R, Holland B, Salmon J (2016). The impact and feasibility of introducing height-adjustable desks on Adolescents' sitting in a secondary school classroom. AIMS public health.

[29] Aminian S, Hinckson EA, Stewart T (2015). Modifying the classroom environment to increase standing and reduce sitting. Building Research & Information.

[30] Demory-Luce D, Morales M, Nicklas T, Baranowski T, Zakeri I, Berenson G (2004). Changes in food group consumption patterns from childhood to young adulthood: the Bogalusa heart study. J Am Diet Assoc.

[31] Golan M, Crow S (2004). Parents are key players in the prevention and treatment of weight-related problems. Nutr Rev.

[32] Collins CE, Watson J, Burrows T (2010). Measuring dietary intake in children and adolescents in the context of overweight and obesity. International journal of obesity (2005).

[33] Harding SK, Page AS, Falconer C, Cooper AR (2015). Longitudinal changes in sedentary time and physical activity during adolescence. The international journal of behavioral nutrition and physical activity.

[34] Busschaert C, De Bourdeaudhuij I, Van Holle V, Chastin SF, Cardon G, De Cocker K (2015). Reliability and validity of three questionnaires measuring context-specific sedentary behaviour and associated correlates in adolescents, adults and older adults. The international journal of behavioral nutrition and physical activity.

[35] Vik FN, Lien N, Berntsen S, De Bourdeaudhuij I, Grillenberger M, Manios Y, Kovacs E, Chinapaw MJ, Brug J, Bere E (2015). Evaluation of the UP4FUN intervention: a cluster randomized trial to reduce and break UP sitting time in European 10-12-year-old children. PLoS One.

[36] Hamar P, Biddle S, Soos I, Takacs B, Huszar A (2010). The prevalence of sedentary behaviours and physical activity in Hungarian youth. Eur J Pub Health.

[37] Hidding LM, Altenburg TM, Mokkink LB, Terwee CB, Chinapaw MJ (2017). Systematic Review of Childhood Sedentary Behavior Questionnaires: What do We Know and What is Next?. Sports medicine (Auckland, NZ).

[38] Kremers SP (2010). Theory and practice in the study of influences on energy balance-related behaviors. Patient Educ Couns.

[39] Kremers SP, de Bruijn GJ, Visscher TL, van Mechelen W, de Vries NK, Brug J (2006). Environmental influences on energy balance-related behaviors: a dual-process view. The international journal of behavioral nutrition and physical activity.

[40] De Clercq B, Pfoertner TK, Elgar FJ, Hublet A, Maes L (2014). Social capital and adolescent smoking in schools and communities: a cross-classified multilevel analysis. Social science & medicine (1982).

[41] Ridley K, Ridgers ND, Salmon J (2016). Criterion validity of the activPAL and ActiGraph for assessing children's sitting and standing time in a school classroom setting. The international journal of behavioral nutrition and physical activity.

[42] Ridgers ND, Timperio A, Cerin E, Salmon J (2015). Within- and between-day associations between children's sitting and physical activity time. BMC Public Health.

[43] Cain KL, Sallis JF, Conway TL, Van Dyck D, Calhoon L (2013). Using accelerometers in youth physical activity studies: a review of methods. J Phys Act Health.

[44] Fereday J, Muir-Cochrane E (2006). Demonstrating rigor using thematic analysis: a hybrid approach of inductive and deductive coding and theme development. Int J Qual Methods.

[45] Mansoubi M, Pearson N, Biddle SJ, Clemes SA (2016). Using sit-to-stand workstations in offices: is there a compensation effect?. Med Sci Sports Exerc.

[46] Atkin AJ, Gorely T, Clemes SA, Yates T, Edwardson C, Brage S, Salmon J, Marshall SJ, Biddle SJ (2012). Methods of measurement in epidemiology: sedentary behaviour. Int J Epidemiol.

[47] De Decker E, De Craemer M, Santos-Lozano A, Van Cauwenberghe E, De Bourdeaudhuij I, Cardon G (2013). Validity of the ActivPAL and the ActiGraph monitors in preschoolers. Med Sci Sports Exerc.

[48] Schrack JA, Cooper R, Koster A, Shiroma EJ, Murabito JM, Rejeski WJ, Ferrucci L, Harris TB (2016). Assessing daily physical activity in older adults: unraveling the complexity of monitors, measures, and methods. J Gerontol A Biol Sci Med Sci.

[49] Wick Katharina, Faude Oliver, Manes Susanne, Zahner Lukas, Donath Lars (2018). I Can Stand Learning: A Controlled Pilot Intervention Study on the Effects of Increased Standing Time on Cognitive Function in Primary School Children. International Journal of Environmental Research and Public Health.

[50] Silva DR, Minderico CS, Pinto F, Collings PJ, Cyrino ES, Sardinha LB**:** Impact of a classroom standing desk intervention on daily objectively measured sedentary behavior and physical activity in youth. *Journal of science and medicine in sport* 2018. 10.1016/j.jsams.2018.01.007 29409737

[51] Erwin H, Beighle A, Routen A, Montemayor B (2018). Perceptions of using sit-to-stand desks in a middle school classroom. Health Promot Pract.

[52] Mehta RK, Shortz AE, Benden ME (2015). Standing Up for Learning: A Pilot Investigation on the Neurocognitive Benefits of Stand-Biased School Desks. International journal of environmental research and public health.

